# Bioactive Compounds of Raspberry Oil Emulsions Induced Oxidative Stress via Stimulating the Accumulation of Reactive Oxygen Species and NO in Cancer Cells

**DOI:** 10.1155/2021/5561672

**Published:** 2021-06-08

**Authors:** Magdalena Grajzer, Benita Wiatrak, Tomasz Gębarowski, Aleksandra Boba, Edward Rój, Daiva Gorczyca, Anna Prescha

**Affiliations:** ^1^Department of Food Science and Dietetics, Wroclaw Medical University, Borowska 211, Wroclaw 50-556, Poland; ^2^Department of Pharmacology, Wroclaw Medical University, Mikulicza-Radeckiego 2, Wroclaw 50-345, Poland; ^3^Department of Basic Medical Sciences, Wroclaw Medical University, Borowska 211, Wroclaw 50-556, Poland; ^4^Department of Genetic Biochemistry, Faculty of Biotechnology, University of Wroclaw, Przybyszewskiego 63/77, Wrocław 51-148, Poland; ^5^Supercritical Extraction Department, New Chemical Syntheses Institute, Al. Tysiaclecia Panstwa Polskiego 13a, Pulawy 24-110, Poland; ^6^3rd Department and Clinic of Paediatrics, Immunology and Rheumatology of Developmental Age, Wrocław Medical University, Koszarowa 5, Wrocław 51-149, Poland

## Abstract

There are growing interests in the complex combinations of natural compounds that may advance the therapy of cancer. Such combinations already exist in foods, and a good representative is seed oils. Two raspberry oils: cold pressed (ROCOP) and one extracted by supercritical CO_2_ (ROSCO2) were evaluated for their chemical characteristics and oil emulsions for cell suppression potential against colon adenocarcinoma (LoVo), doxorubicin-resistant colon adenocarcinoma (LoVo/DX), breast cancer (MCF7), doxorubicin-resistant breast cancer (MCF7/DX), and lung cancer (A549) cell lines. The cytotoxicity was also assessed on normal human dermal fibroblasts (NHDFs). With increasing concentration of raspberry oil emulsions (0.5–10%), increasing inhibition of cancer cell viability and proliferation in all of the lines was observed, with different degrees of potency between cancer types and oil tested. ROSCO2 strongly induced free radical production and DNA strand damage in LoVo and MCF7 cells especially doxorubicin-resistant lines. This suggests that ROSCO2 engages and effectively targets the vulnerabilities of the cancer cell. Generally, both ROSCO2 and ROCOP could be a nontoxic support in therapy of selected human cancers.

## 1. Introduction

Cancer is the leading cause of death in the world. And yet, a growing problem in clinically effective chemotherapeutic treatment is the increased resistance to commonly used cytostatics. Treatment of patients with chemotherapy becomes ineffective and often only results in the side effects of cytostatics and disease progression. Interests of many researches have had focused recently on compounds that are capable of stimulating reactive oxygen species (ROS) and reactive nitrogen species (RNS) formation, which may collectively be responsible for damaging cells, including cancer [1–3]. These compounds are often grouped in complex combinations, acting synergistically, and may modestly advance the therapy of cancer. Such combinations already exist in foods, and a good representative is seed oils. In recent years, oils recovered from various seeds have been an unconventional lipid source occupying an important position in the nutrition field and extensively evaluated regarding their effects on health. Vegetable seed oils are a nutrition source of polyunsaturated fatty acids, sterols, and tocopherols, but also consist of other bioactive compounds presented in smaller quantities: carotenoids and phenolics. Raspberry seed oil is an excellent source of polyunsaturated fatty acids (PUFAs) exceeding 80% of the total lipid content and various micronutrients of high bioactivity [4–7].

Pharmacological properties of raspberry seed oils have been scrutinized, with anti-inflammatory, antioxidative, and hypolipidemic effects in animal studies [8, 9]. Antioxidant properties of raspberry oil may also suggest their chemopreventive activities. The primary fatty acids are linoleic acid (LA) and *α*-linolenic acid (ALA), which are present in a ratio of less than two to one. Oleic acid in another significant fatty acid and in raspberry oil is present at 10-12%. Several mechanisms seem to be involved in the antitumor PUFA activity, including alterations in cell signalling, chromatin remodeling, and DNA methylation [10–12]. In addition to PUFA and monounsaturated fatty acids (MUFAs), raspberry seed oils contain high levels of phytosterols (up to 5384 mg/kg), tocopherols (up to 4000 mg/kg), and carotenoids (ca. 25 mg/kg), higher than many other berry species [4, 5, 7]. Among the bioactivities of minor components of raspberry seed oil, the important role of tocopherols in the protection of cell membranes from oxidation, gene regulation, and signal transduction has been well documented [13, 14]. *In vitro* studies have shown the effects of *γ*- and *α*-tocopherols in reducing the activity of colon cancer and breast cancer cells, respectively [14]. Previous studies have identified specific antioxidant properties of carotenoids. Both nonoxygenated carotenoids and the oxygenated derivatives xanthophylls, singly and in combination, may suppress cancer development and improve the immune response [15–17]. Sterols present in raspberry oil have shown anti-inflammatory, anticarcinogenic, and wound-healing properties [18]. However, the biological activity of raspberry seed oil with a view to different oil recovery techniques has not been extensively investigated.

Raspberry seeds are a decent oil source; 10-25% of the oil can be recovered. The yield and composition of oil and consequently its biological properties are influenced by the method of oil extraction. Among oil extraction technologies, cold pressing is a widely adopted practice in manufacturing high-quality and environmentally friendly oil. The limitation of cold pressing is low yield and exposing oil rich in PUFA to oxidation. Supercritical CO_2_ (SCO2) oil extraction has attracted researchers' attention due to better oil recuperation from residues, the absence of oxygen, and operating at low temperature that can minimize oxidative oil deterioration. Another important asset of supercritical CO_2_ is the high penetration and solvating power. In the literature, the authors have highlighted that SCO2 extraction is selective towards nonpolar and low molecular weight nonpolar and slightly polar components such as phytosterols, tocopherols, and phenols [19–21], not to mention the full CO_2_ retrievability, which makes the oil extraction environmentally friendly [19].

Taking together all the facts quoted beforehand, this study is primarily aimed at characterizing raspberry oils obtained by supercritical CO_2_ extraction and cold pressing and justifying their cell suppression potential against a panel of human normal and cancer cell lines. These findings would provide the basis for further *in vivo* studies as well as for clinical trials.

## 2. Materials and Methods

### 2.1. Raw Material

**Figure 1:**
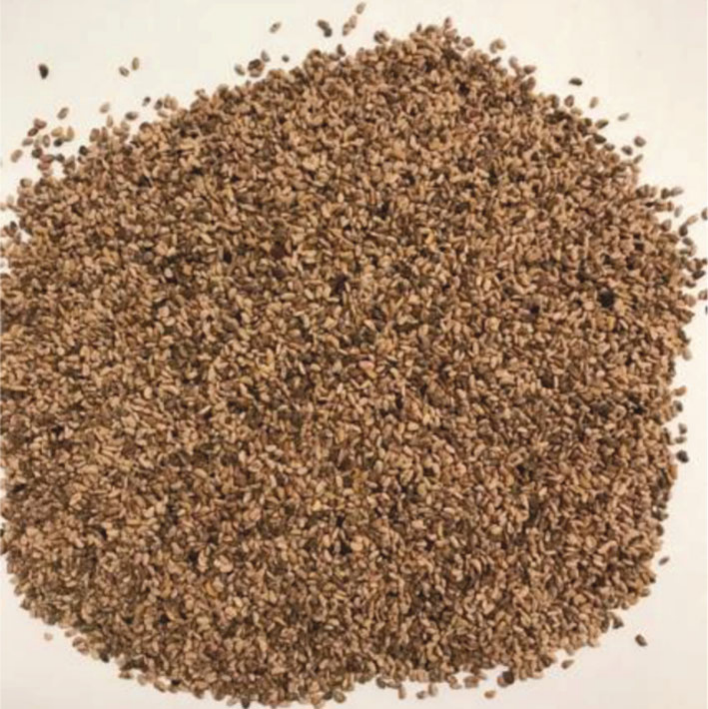
Raspberry seeds used in cold pressing and supercritical extraction came from the same batch.

Seeds provided by the Polish manufacturer Oleowita (Milicz, Poland) were purified from any undesirable remains and then freeze-dried (Figure 1). Both methods of oil recovery seeds came from the same harvest.

### 2.2. Seed Oils

Raspberry oil obtained by supercritical carbon dioxide extraction (ROSCO2) was obtained from seeds crushed in a mill crusher under nitrogen to particle sizes of approximately 0.4–0.6 mm immediately before supercritical carbon dioxide extraction. The supercritical carbon dioxide extraction was carried out on an extraction apparatus pilot plant (ELAB) designed and made by engineers from the Łukasiewicz Research Network–New Chemical Synthesis Institute in Pulawy (Poland). 1660 g of ground seeds was loaded into the extractor basket. The extraction procedure has been described in detail elsewhere [21]. Briefly, the extraction with supercritical carbon dioxide started at a pressure of 340 bar and temperature of 40°C. The flow rate and CO_2_ consumption were 89.7 kg CO_2_/h. and 62.0 kg CO_2_/kg, respectively. The extraction yield was 10.65 wt.%.

Cold-pressed raspberry oil (ROCOP) was obtained by cold pressing in a hydraulic press under nitrogen atmosphere by the Polish manufacturer Oleowita and immediately (within 24 hours) delivered to the laboratory. The extraction yield was 9 wt.%.

Both oils were bottled up under nitrogen atmosphere and stored at -26°C until analysis.

### 2.3. Oils' Chemical Characteristics

#### 2.3.1. Fatty Acid Compositions

The procedure of fatty acid determination was described previously by Prescha et al. [22]. The chromatographic separation was performed on a gas chromatograph 6890 N (Agilent Technologies, USA) equipped with an FID detector and a Supelco SPTM-2560 fused silica capillary column 100 m × 0.25 mm × 0.2 *μ*m (Bellefonte, USA). Hydrogen at a flow rate of 1.5 ml/min was used as a carrier gas, and the initial temperature of chromatographic separation was 165°C held for 10 min; then, the temperature was increased at the rate of 2°C/min to the final temperature of 220°C held for 10 min. The identification of fatty acids was accomplished using external fatty acid methyl ester (FAME) standards.

#### 2.3.2. Phytosterol and Squalene Content

The analysis of sterols and squalene in oil samples was performed according to the protocol described by Shukla et al. [23]. Volatile components were separated on a DB-5MS capillary column 30 m × 0.25 mm × 0.1 *μ*m (J & W Scientific, USA) using a 7890A gas chromatograph equipped with an electron ionization source and the autosampler system PAL3 RSI (CTC Analytics AG, Switzerland) coupled to a 5975C mass spectrometer (Agilent Technology, USA). Helium at a flow rate of 1.1 ml/min was used as the carrier gas. Source temperature was 230°C, and temperature of the transfer line was 290°C; the separation started at 120°C held for 1 minute; then, the temperature was increased at the rate of 5°C/min to a final temperature of 290°C held for 15 minutes. Identification of individual phytosterols and squalene was carried out using mass spectra and retention times of external standards.

#### 2.3.3. Tocopherol and Carotenoid Composition and Content

The procedures of extraction of tocopherols and carotenoids from oil samples and analysis were based on the method already described by Fromm et al. [24]. Then, the unsaponifiable fraction was separated on a C18 UPLC column (Acquity, 100 mm × 2.1 mm × 1.7 *μ*m, Waters, USA) connected to an Acquity UPLC system equipped with a PDA detector (Waters, USA). Two eluents were used as follows: (a) 30% methanol, 50% acetonitrile, and 20% water; (b) 50% methanol and 50% acetonitrile (v/v) at a flow rate of 0.5 ml/min in a timed gradient program: 100% A held for 1 min, linear gradient up to 100% B for 4 min, from 5 min to 10 min 100% B.

#### 2.3.4. Phenolic Compounds' Compositions and Content

The procedure of phenolic compounds' extraction was acquired from the protocol previously published [25] using solid-phase C8 cartridges. Then, the hydrophilic compounds were separated on a C18 UPLC column (BEH Acquity, 100 mm × 2.1 mm × 1.7 *μ*m, Waters, USA) connected to an Acquity UPLC system equipped with PDA 200–500 nm and mass spectrometer Xevo-Q-TOF (Waters, Milford, CT, USA). The mobile phase was a mixture of A—deionized waterformic acid (99.5 : 0.5), and B—acetonitrileformic acid (99.5 : 0.5), at a flow rate of 0.5 ml/min. The electrospray ionization source of the mass spectrometer operated at a capillary voltage of ±2.80 kV in the positive and negative ion mode, respectively, a sampling cone of 66 kV, and an extraction cone of 4.0 kV. Collision energy was set at 0, 20, 20–30, 30, and 30–50 kV. Data were processed using MassLynx 2.0 (Waters, Milford, CT, USA). Single components were identified by comparison of experimental mass, UV absorption spectra, and retention time with standards and literature data.

#### 2.3.5. Statistical Analysis

The statistical significance of differences in the content of oil components was determined using the *t*-test and Mann-Whiney *U* test for normally and not normally distributed variables, respectively. The normality of the variable distribution was assessed with the Shapiro-Wilk test.

### 2.4. *In Vitro* Studies

#### 2.4.1. Preparation of Oil Emulsions

For *in vitro* studies, the raspberry oil emulsions were formulated in accordance with a protocol developed in a study by Skorkowska-Telichowska et al. [26]. One gram of soybean lecithin, 0.5 ml of Tween 80, and 2.5 ml of oil were mixed gradually and completed with a 5 ml of an aqueous phase containing glycerol 25% v/v. Raspberry oil emulsions were prepared at 0.5%, 1%, 2%, 5%, and 10% at room temperature (RT) and in sterile conditions.

#### 2.4.2. Cell Lines and Culture Conditions

All cell lines were maintained under sterile conditions at 37°C in a humidified atmosphere of 5% CO_2_ in the complete culture medium. Normal human dermal fibroblasts (NHDFs; Lonza: CC-2511) and five cancer lines—colon adenocarcinoma cell line (LoVo; ECACC: 870601011-1VL), doxorubicin-resistant colon adenocarcinoma cell line (LoVo/DX), breast cancer (MCF7; ECACC: 86012803-1VL), doxorubicin-resistant breast cancer (MCF7/DX), and lung cancer (A549; ECACC: 86012804-1VL)—were cultured in an appropriate medium supplemented with penicillin (10000 U/ml), streptomycin (10 mg/ml), L-glutamine (200 mM), and 10% fetal bovine serum (FBS). NHDF was purchased from Lonza (Verviers, Belgium; cat. no. CC-2511) and cultured in Dulbecco's Modified Eagle Medium (DMEM) without phenol red. The colon adenocarcinoma cell line (LoVo) was obtained from EATCC (cat. no. 870601011-1VL), and doxorubicin-resistant breast cancer (LoVo/DX) was derived from them as a result of incubation in the presence of low concentrations of doxorubicin for a period of three months [2]. Both types of cell line were grown in Dulbecco's Modified Eagle Medium/Nutrient Mixture F-12 (DMEM/F-12) medium. The breast cancer line (MCF7) was purchased from the EATCC collection (cat. no. 86012803-1VL), and the doxorubicin-resistant breast cancer line (MCF7/DX) was derived from it as a result of incubation in the presence of low concentrations of doxorubicin for a period of three months [27]. The lung cancer line (A549) was purchased from EATCC (cat. no. 86012804-1VL). Cells of both breast cancer lines and lung cancer were cultured in Minimal Essential Medium (MEM). The cells were evaluated at least twice a week under a microscope and then passaged using TrypLE solution or the appropriate medium was changed.

When the cell confluence was above 70%, the cells were separated from culture flasks using TrypLE. The cell suspension was collected into centrifuge tubes, and an appropriate medium for the cell line was added to inactivate TrypLE activity. The tubes were then centrifuged at 1000 × g for 5 min. At this time, the number of cells was counted in a Bürker chamber.

A negative control was used in this study as a reference, which was a cell culture incubated in the primary medium without tested oils. All assays were performed in five independent replicates.

#### 2.4.3. Cell Viability Assay

The methylthiazol tetrazolium (MTT) assay, based on the conversation of 3-(4,5-dimethylthiazol-2-yl)-2,5-diphenyltetra-zolium bromide to MTT formazan, was used to evaluate the effect of oils' emulsions on cell viability according to the ISO 10.9993 standard, part V. For the assays' purpose, cells were seeded on 96-well plates at a density 1 × 10^4^ cells/ml per well and subjected to 24-hour incubation under 5% CO_2_ in air. Then, the cells were treated with increasing concentrations of oil emulsion. At the end of the incubation time, the supernatant was removed, then the cells were washed with PBS, and 50 *μ*l of 1 mg/ml MTT solution in MEM without phenol red was added to the culture plates and left in a CO_2_ incubator at 37°C for 2 hours. The supernatant was then removed, and colored formazan was added to the medium, after being dissolved in 100 *μ*l of isopropanol for 30 min while shaking. The percentage of viable cells was calculated by measuring the absorbance of the colored formazan reaction product at 570 nm using a Varioskan LUX microplate reader (Thermo Scientific, USA).

#### 2.4.4. Cell Proliferation Assay

Cell proliferation was determined by the sulforhodamine B assay (SRB). Cells were seeded on 96-well plates at a density of 1 × 10^4^ cells/ml per well. Before the addition of the oil emulsions, one plate from each cell line was fixed with cold 50% trichloroacetic acid (TCA) at 4-8°C (these plates were the control). After 48-hour incubation with increasing concentrations of oil emulsion, the culture plates were also fixed with 50% TCA for 1 h at 4-8°C. The plates were then washed five times under running water, the plates were air-dried, and then, sulforhodaminen B dye was added for another 30 minutes to stain cell proteins. Unbound dye was removed five times by washing with 1% acetic acid and air-dried again. The SRB dye was dissolved in Trizma solution, and the absorbance was measured at 570 nm, using a Varioskan LUX microplate reader (Thermo Scientific, USA).

#### 2.4.5. Scavenging Activity against Reactive Oxygen Species (ROS)

The spectrofluorometric method using 2′,7′-dichlorofluorescein-diacetate (DCF-DA) for quantifying the intracellular levels of reactive oxygen species (ROS) produced in five cultured cancer cell lines was utilized. Cells were seeded on 96-well plates at a density 1 × 10^4^ cells/ml per well. After 24-hour incubation with oil emulsion, the supernatant was transferred to new plates, and the DCF-DA solution was added to the culture for the next 1 hour of incubation. After that time, the supernatant was removed, cells were washed twice with phosphate buffered saline (PBS), and then, 100 *μ*l of H_2_O_2_ was added for the next 30 min. Finally, the fluorescence was measured at 𝜆_ex_. =485 nm and 𝜆_em_. =535 nm using a Varioskan LUX microplate reader (Thermo Scientific, USA) [28].

#### 2.4.6. Scavenging Activity against Nitric Oxide

For the evaluation of intracellular concentration of nitric oxide, Griess reagent (1 : 1 mixture (v/v) of 1% sulfanilamide in 5% phosphoric acid and 0.1% N-(1-naphthyl)ethylenediamine dihydrochloride) was added to the supernatant plates. The plates were left for 20 minutes in the dark at RT. Nitrite level was measured at 548 nm using a Varioskan LUX microplate reader (Thermo Scientific, USA) [29].

#### 2.4.7. Fast Halo Assay

The fast halo assay (FHA) was conducted to evaluate the effect of the tested oils on double-strand breaks (DSBs). Cells were seeded on 24-well plates at a density 2.5 × 10^4^ cells/ml per well. After incubating with the tested oils, the supernatant was transferred to centrifuge tubes, which were previously properly signed and prepared, and the culture was washed with PBS, which was also collected into tubes. The cells were separated from the surface of the plate with TrypLE solution up to 3 min at 37°C in a CO_2_ incubator, and the cell suspensions were transferred into tubes, which were then centrifuged at 1000 × g for 5 min. After removal of the supernatant, the cell pellet was washed in PBS and again centrifuged at 1000 × g for 5 min. The cells were resuspended in PBS with Ca^2+^ and Mg^2+^ at a density of 1000 cells/*μ*l; the cells in tubes were placed in a water bath. Then, 120 *μ*l of 1.25% low melting agarose in PBS was added to the cells, and the mixture was immediately squeezed between a slide coated with agarose (high melting point) and a coverslip. The slides were placed on a cooling block for 10 min for gel formation. The coverslips were then removed, and the slides were placed in the lysis buffer overnight. The slides were then transferred into alkaline solution (pH = 13.0) for 30 min and then washed twice for 5 min in neutralizing buffer. The preparations were stained using 5 *μ*M DAPI for 20 min and examined immediately under a fluorescence microscope. Photographs were taken, and then, the ratio of cell nucleus diameter to halo diameter (which is a measure of DNA damage) was analyzed [29].

#### 2.4.8. Statistical Analysis

The results obtained in the MTT and SRB assays are presented as IC_50_ values, which indicate the concentrations of the oil emulsions at which 50% inhibition of succinate dehydrogenase activity (MTT assay) or total cellular protein synthesis (SRB assay) was observed (depending on the assay performed) compared to the control. The normality of the data and the equality of variance were checked using the Shapiro-Wilk and Levene'a tests, respectively. Due to the possibility of adopting the hypothesis of normal distribution and equal variance, parametric tests were used for statistical analysis. Other results are shown as mean ± SEM (standard error of the mean) relative to the control (*E*/*E*_0_). *E* represents the result obtained for the culture with the test oils after subtraction of the values obtained for the solvent alone, and *E*_0_ is the result for negative control.

The two-way ANOVA was used (with Tukey post hoc test) for the comparison of data obtained for cell lines. In all assays, *p* < 0.05 was used as the significance level. To compare the activity of the tested oils, multiple-criteria decision analysis (MCDA) was carried out using a weighted sum model (WSM). The weights were chosen according to the meaning of each biological assay. Pearson's correlation coefficients were calculated to show the relationship between double-stranded DNA breaks and free radical levels.

## 3. Results

To compare the impact of bioactive compounds of oils' emulsions on cell treatment, several chemical characteristics were measured.

### 3.1. Oils' Chemical Characteristics

Raspberry oil extracted by supercritical CO_2_ (ROSCO2) contained about 78% PUFA, and in comparison with ROCOP—raspberry oil cold-pressed, this was about 18% more. In both raspberry oils, LA represented slightly more than 50% of total fatty acids, and ALA represented around 27% of ROSCO2. However, in ROCOP, 2.5 times less ALA was recorded than in ROSCO2. Oleic acid (OA) is another significant fatty acid present at 15% in ROSCO2 and 26% in ROCOP.

In addition to high levels of PUFA, raspberry oil contained considerable amounts of phytosterols, reaching over 20 g/kg in ROSCO2, while in ROCOP, the total phytosterol content was only ca. 30% of that determined in ROSCO2 (Table 1). The primary component of the phytosterol fraction in both oils was *β*-sitosterol, which accounted for 75% in ROSCO2 and 67% in ROCOP, and other significant phytosterols present in smaller amounts were *Δ*^5^-avenasterol and 24-methylenecycloartenol (10-11% and 6-7%, respectively).

Squalene content in both oils was low, and in ROSCO2, it was higher than in ROCOP by ca. 12%. *β*-Amyrin content was recorded as 1.8-fold higher in ROCOP that in ROSCO2.

The total amount of tocopherols was 1105 mg/kg in ROCOP, and it was 3 times less than the total amount of tocopherols obtained in this study for ROSCO2. In both oils, *γ*-tocopherol was the dominant one but in ROSCO2, its contribution to the total tocopherols was about 10% higher than that recorded for ROCOP.

Raspberry oil obtained by SCO2 extraction stood out in terms of carotenoid content. The total carotenoid content in ROSCO2 was almost ten times higher than that in ROCOP. Moreover, SCO2 extraction seemed to recover better xanthophylls, especially all-transzeaxanthin, and carotenes.

Phenolic compound recovery in raspberry oils was again higher in ROSCO2 than ROCOP, but still was not so pronounced as it was in the case of carotenoid content or composition. The difference in total phenolic content was 6 times greater. ROSCO2 was characterized by a pronounced presence of vanillin, 4-hydroxybenzaldehyde, and 4-hydroxybenzoic acid.

### 3.2. *In Vitro* Studies

#### 3.2.1. Cytotoxicity

**Figure 2:**
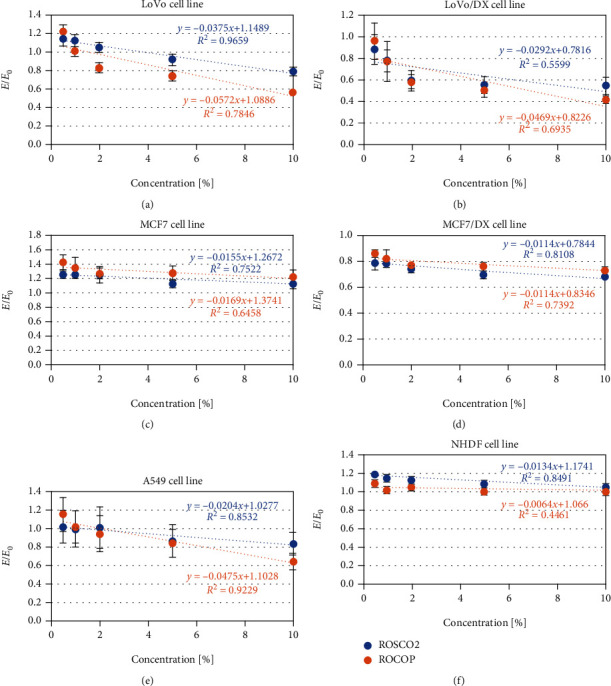
The effect of oil emulsion from raspberry oils extracted by supercritical CO_2_ (ROSCO2) and cold pressed (ROCOP) at various concentrations—0.5%, 1%, 2%, 5%, and 10%—on the viability of colon adenocarcinoma cell lines (LoVo), doxorubicin-resistant colon adenocarcinoma cell line (LoVo/DX), breast cancer cell line (MCF7), doxorubicin-resistant breast cancer cell line (MCF7/DX), lung cancer cell line (A549), and normal human dermal fibroblast (NHDF). The results were compared to control and expressed as *E*/*E*_0_—the ratio of spectrometrically measured viability of cells with tested oils (*E*) to a negative control culture (*E*_0_—cells without tested oils). Linear regression models and coefficients of determination were calculated.

In the entire range of concentrations tested for both oils, no significant reduction in metabolic activity (MTT assay) in normal cells was observed—IC_50_ values were high (51.9% and 88.4%, respectively, for ROSCO2 and ROCOP oils) (Figure 2 and Table 2).

In LoVo cancer cells, ROCOP oil had a stronger effect, while in MCF7/DX cells, ROSCO2 oil had slightly but significantly more affected cells' metabolic activity. Generally, for both oils, a greater reduction of metabolic activity was observed in doxorubicin-resistant cell lines, both colorectal and breast cancer. Based on the calculated IC_50_ values, it can be concluded that in the lung cancer line, ROCOP oil inhibited metabolic activity approximately twice as strongly as ROSCO2.

#### 3.2.2. Cell Proliferation

**Figure 3:**
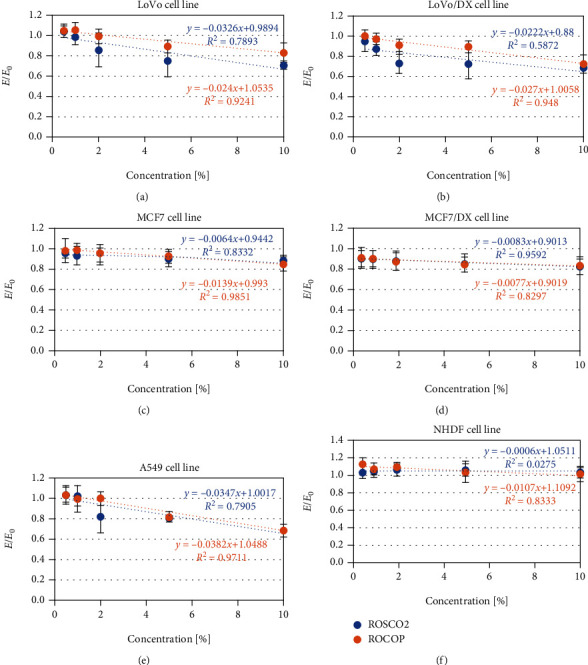
*In vitro* antiproliferative activity of oil emulsion from raspberry oils extracted by supercritical CO_2_ (ROSCO2) and cold pressed (ROCOP) against human cancer and normal cell lines. Antiproliferative effect of oil emulsions in 0.5%, 1%, 2%, 5%, and 10% concentrations on colon adenocarcinoma cell line (LoVo), doxorubicin-resistant colon adenocarcinoma cell line (LoVo/DX), breast cancer cell line (MCF7), doxorubicin-resistant breast cancer cell line (MCF7/DX), lung cancer cell line (A549), and normal human dermal fibroblast (NHDF). Cell growth percentage was analyzed with sulforhodamine B (SRB) assay. The results were compared to control and expressed as *E*/*E*_0_. *E*—cells with tested oils; *E*_0_—a negative control culture (cells without tested oils). Linear regression model and coefficients of determination were calculated.

Raspberry oil (ROSCO2 and ROCOP) emulsions were examined for their antiproliferative activity against a panel of human cancer (5) and normal (1) cell lines. ROSCO2 emulsions exhibited a noncytotoxic effect on human normal fibroblast cells (NHDFs) at all studied concentrations. In contrast to ROSCO2, the ROCOP emulsion showed an antiproliferative effect at 57.0% (IC_50_) (Figure 3 and Table 3). However, the different cancer cell lines exhibited different sensitivity to raspberry oil emulsions, evident from the different IC_50_ values (Table 3). In the case of colorectal cancer cells (LoVo), ROSCO2 oil was more cytotoxic than ROCOP, but at the same time, the effect was slightly weaker for the doxorubicin-resistant line (LoVo/DX). In contrast to the doxorubicin-sensitive breast cancer cell line (MCF7), where stronger antiproliferative activity was demonstrated for ROCOP oil, in the doxorubicin-resistant MCF7 cells, the effect was similar for both oils. For the lung cancer line (A549), the IC_50_ values determined for both oils were almost identical.

#### 3.2.3. Intracellular Levels of Free Oxygen Radicals and Nitric Oxide

To assess the effect of the tested oils on the level of free oxygen radicals and nitric oxide (NO), DCF-DA and Griess assays were performed, respectively. Some cytostatics increase the level of free radicals in cancer cells, which in turn causes DNA damage and subsequent cancer cell death. At the same time, the observed ROS levels are lower in resting cytostatic resistant cell lines compared to the corresponding sensitive cell lines [2].

**Figure 4:**
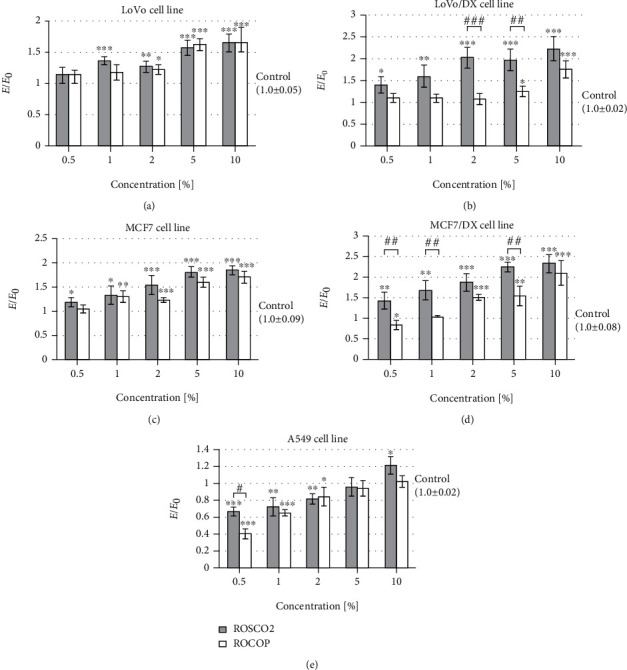
Level of free oxygen radicals after incubation with oil emulsion from raspberry oils extracted by supercritical CO_2_ (ROSCO2) and cold pressed (ROCOP); ^∗^*p* < 0.05, ^∗∗^*p* < 0.01, and ^∗∗∗^*p* < 0.001—significant difference compared to control; ^#^*p* < 0.05, ^##^*p* < 0.01, and ^###^*p* < 0.001—significant difference between tested oils. The results were compared to control and expressed as *E*/*E*_0_—the ratio of spectrofluorometrically measured level of free oxygen radicals of cells with tested oils (*E*) to a negative control culture (*E*_0_—cells without tested oils). Cell lines: colon adenocarcinoma (LoVo), doxorubicin-resistant colon adenocarcinoma (LoVo/DX), breast cancer (MCF7), doxorubicin-resistant breast cancer (MCF7/DX), and lung cancer (A549).

This study showed significant higher ROS levels in both doxorubicin-resistant cancer cell lines (colorectal and breast) compared to the corresponding sensitive cell lines after incubation with tested raspberry oils, especially ROSCO2 (the increase was 20-80% in colorectal cancer cell and 30-60% in breast cancer cells) (Figure 4). A statistically significantly higher level of free oxygen radicals in resistant lines of colorectal and breast cancer after the use of ROSCO2 oil was observed (40-100% for the colon cancer line and 30-70% for breast cancer) when compared to ROCOP oil. However, the impact of the tested oils on the ROS level in doxorubicin-sensitive lines was independent of the method of oil extraction. In turn, after incubation of lung cancer cells with the examined oils in the concentration range of 0.5-5%, a statistically significant reduction in the level of ROS was observed. At the lowest concentration (0.5%), stronger scavenging of free oxygen radicals was observed after treatment of this cell line with ROSCO2 emulsion in comparison to ROCOP. In the case of A549 cells, incubation with a 10% concentration of ROSCO2 oil statistically significantly increased the level of free oxygen radicals.

**Figure 5:**
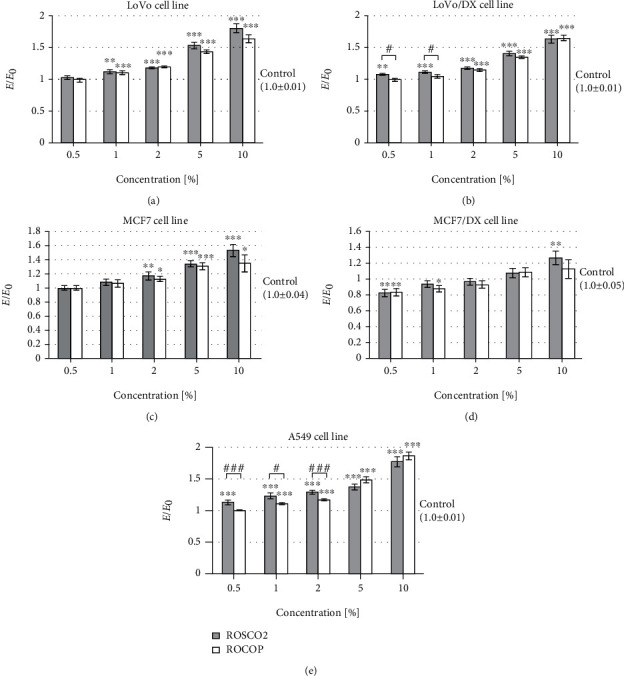
Level of nitric oxide after incubation with oil emulsion from raspberry oils extracted by supercritical CO_2_ (ROSCO2) and cold pressed (ROCOP); ^∗^*p* < 0.05, ^∗∗^*p* < 0.01, and ^∗∗∗^*p* < 0.001—significant difference compared to control; ^#^*p* < 0.05 and ^###^*p* < 0.001—significant difference between tested oils. The results were compared to control and expressed as *E*/*E*_0_—the ratio of spectrometrically measured level of NO of cells with tested oils (*E*) to a negative control culture (*E*_0_—cells without tested oils). Cell lines: colon adenocarcinoma (LoVo), doxorubicin-resistant colon adenocarcinoma (LoVo/DX), breast cancer (MCF7), doxorubicin-resistant breast cancer (MCF7/DX), and lung cancer (A549).

In contrast, nitric oxide levels were higher in the doxorubicin-sensitive LoVo and MCF7 cells than in their resistant counterparts (Figure 5). After incubation with each tested oil, a concentration-dependent increase in NO level in the concentration range of 1-10% was observed in these cell cultures. In the doxorubicin-resistant colorectal cancer line, a statistically significant increase in NO level was observed in the entire tested concentration range after incubation with ROSCO2 emulsion and in the concentration range of 2-10% of ROCOP. The level of nitric oxide in LoVo/DX after administration of ROSCO2 at the concentrations of 0.5 and 1.0% was significantly higher than in the case of ROCOP. In the doxorubicin-resistant cell line of breast cancer, after incubation with oils at concentrations of 0.5-2.0%, a decrease in nitric oxide levels was observed compared to the culture incubated only in medium. In the case of the lung tumor cell line (A549), there was a concentration-dependent increase in NO level after treatment with the tested oils. What is more, in the concentrations of 0.5-2.0%, this increase was statistically significantly higher after using supercritical extracted oil compared to cold-pressed oil.

#### 3.2.4. DNA Double-Strand Damage Assessment

**Figure 6:**
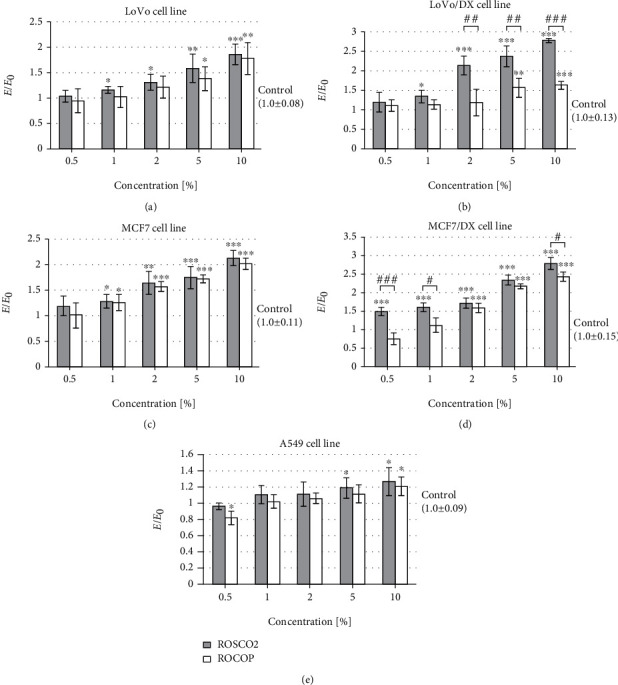
Amount of DNA strand damage after application of the oil emulsion from raspberry oils extracted by supercritical CO_2_ (ROSCO2) and cold pressed (ROCOP); ^∗^*p* < 0.05, ^∗∗^*p* < 0.01, and ^∗∗∗^*p* < 0.001—significant difference compared to control; ^#^*p* < 0.05, ^##^*p* < 0.01, and ^###^*p* < 0.001—significant difference between tested oils. The results were compared to control and expressed as *E*/*E*_0_. *E*—cells with tested oils; *E*_0_—a negative control culture (cells without tested oils). Cell lines: colon adenocarcinoma (LoVo), doxorubicin-resistant colon adenocarcinoma (LoVo/DX), breast cancer (MCF7), doxorubicin-resistant breast cancer (MCF7/DX), and lung cancer (A549).

To investigate the amount of DNA strand damage after application of the tested oils, a fast halo assay (FHA) was performed. In all cancer lines tested, a concentration-dependent increase in DNA strand breaks was observed regardless of the oil used (Figure 6). At the same time, the amount of DNA damage was always greater after the treatment of cell cultures with ROSCO2 than ROCOP, and these differences were statistically significant for some oil concentrations in doxorubicin-resistant cell lines, both LoVo/DX and MCF7/DX.

Correlation coefficients between DNA damage and ROS or NO levels were assessed. In all cases, after using each tested oil (except for ROSCO2 oil in A549 cells), a strong positive and statistically significant correlation between NO level and DNA damage was found (Table 4). A strong positive correlation was also found between the ROS level and DNA strand damage in the LoVo and MCF7/DX lines after incubation with both tested oils and in the MCF7 line after using ROSCO2 oil.

#### 3.2.5. Multiple-Criteria Decision Analysis

**Figure 7:**
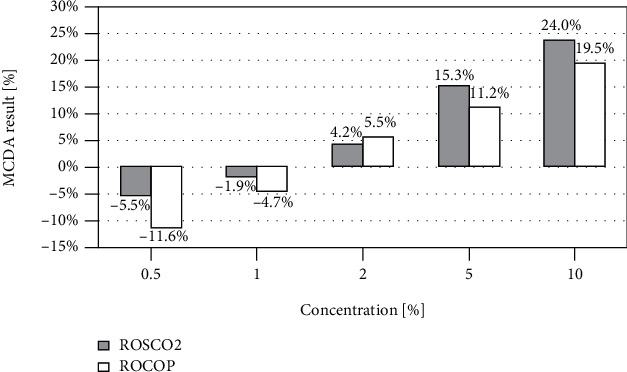
Results of multiple-criteria decision analysis of the collected results of all assays carried out, expressed as the percentage value corresponding to the strength of the positive oil effect; MCDA results greater than zero indicate the expected positive effect on the cancer cell lines.

Multiple-criteria decision analysis (MCDA) was performed based on the results obtained from all assays. The MCDA results showed that at concentrations where the oil effect was clearly pronounced (5 and 10%), the activity of the supercritical extracted oil was stronger compared to the cold-pressed oil (Figure 7). At a concentration of 2%, the effect of the oils was very similar. On the other hand, low concentrations showed the opposite activity than expected, and it was stronger in the case of ROCOP oil. Generally, it can be concluded that in the concentration range of 2-10%, the tested oils showed a concentration-dependent suppressive impact on cancer cells.

## 4. Discussion

The overall objective of this study was to determine whether raspberry oils obtained by different extraction techniques have any diverse and selective effects against the panel of human normal and cancer cell lines. From the current results, it is clear that the two raspberry oils—extracted by supercritical CO_2_ and cold pressed—significantly differ in phytochemical characteristics. In comparison to cold-pressed raspberry oil (ROCOP), SCO2-extracted raspberry oil stood out in terms of composition of fatty acids (particularly ALA), phytosterols (substantially *β*-sitosterol, *Δ*5-avenasterol, and 24-methylene-cycloartenol), tocopherols, carotenoids, and phenolic compounds. In both raspberry oils, the fatty acid composition was close to the data published so far [5–7, 30], except for ALA and OA in ROCOP, the levels of which were 2- to 3-fold lower than the results previously published [5, 30].

From the proceeding results, it is clear that both raspberry oils exert multiple *in vitro* suppressive effects on the human colon adenocarcinoma cell line (LoVo), doxorubicin-resistant colon adenocarcinoma cell line (LoVo/DX), breast cancer cell line (MCF7), doxorubicin-resistant breast cancer cell line (MCF7/DX), and lung cancer cell line (A549), but its cancer cell suppression activity was equally complex. However, the differences in the content of bioactive components of both oils reflected in a distinct cancer-suppressive potential.

The studied oils differ in terms of polyunsaturated fatty acid (PUFA) composition and the ratio of n-6/n-3 PUFA (mainly LA/ALA), which in turn could considerably affect tumor cells. The cancer cell suppression activity of LA and ALA is not definite as yet [31]. It has been previously shown that the action of LA on cancer growth depends on its concentration and the type of cancer cell tested. Lu et al. [32] observed cancer cell suppression at high LA concentration in LoVo by MTT assay. This could explain the stronger inhibition of mitochondrial metabolite activity by ROCOP, which is rich in LA, in comparison to ROSCO2. As the reactive oxygen species formation in LoVo cell lines was again more pronounced in ROCOP than ROSCO2, especially in the highest range of concentrations, this may indicate that LA-induced cell death was primed by the mitochondrial apoptotic pathway [33]. Thus, the mechanisms underlying ROCOP apoptotic activity may enhance the cellular oxidant status including mitochondrial dysfunction [32]. Abundant in ALA, ROSCO2 also affected considerably cell viability in LoVo and LoVo/DX cell lines in dose-dependent manners and moreover was more effective than ROCOP in proliferation inhibition of LoVo cells. Apoptotic cell activation via induction of caspase activity, both extrinsic and intrinsic pathways, was previously observed for ALA [10, 11]. These findings also enhance the understanding of the n-6/n-3 ratio in chemoprevention of colon and breast cancers. It seems that smaller ratios could be more favorable in doxorubicin-resistant breast cancer than colon cancer. The observed correlation is in line with previous research where flaxseed oil rich in ALA reduced growth and increased apoptosis in several breast cancer lines, dynamically affecting gene expression and modifying signalling pathways [34].

Polyunsaturated fatty acids may influence cancer cell membrane integrity, facilitating the penetration of other potentially cancer toxic molecules. This means that PUFAs are not solely or even primarily responsible for the apoptosis of cancer cell lines observed in raspberry oil emulsions. The raspberry oils contained a pronounced amount of phytosterols and an interesting compound derivative from 4,4-dimethylphytosterols. 4,4-desmethylphytosterols exhibit anticancer effects in many cancers including skin cancer, bladder cancer, and colon cancer [18, 35]. The substantially higher amount of *β*-sitosterol and 4,4-desmethylphytosterols may contribute to the strong antiproliferative effect of ROSCO2 against LoVo and LoVo/DX cells. Tocopherols were also presented in greater concentrations in ROSCO2, especially *γ*-tocopherol, a strong apoptotic natural compound [14]. An even stronger proapoptotic effect than *γ*-tocopherol is exhibited by *δ*-tocopherol, presented in greater concentration in ROSCO2 than ROCOP [36]. Both of these isoforms activate peroxisome proliferator-activated receptor *γ* (PPAR*γ*) expression, which is known to control inflammation by inducing apoptosis and inhibiting cancer cell proliferation [37, 38]. The *γ* isoform shows a similar cytotoxic effect as n-3 PUFA. In contrast, *α*-tocopherol is a weaker proapoptotic inducer. Some studies have revealed that this homolog may attenuate the cell cycle block induction as well as the cytotoxic and proapoptotic effects of n-3 fatty acids on cancer cells [39]. *α*-Tocopherol has also been shown to compromise the cytotoxic and cytostatic action of chemotherapeutic agents acting as protein kinase inhibitors. In prostate and colon cancer cells, intracellular concentrations increased as the concentration of tocopherols added to the media was increased. This effect was more pronounced in cells treated with *γ*-tocopherol than those treated with *α*-tocopherol [14]. Interestingly, the ratio of *α* : *γ* : *δ* tocopherols found in this study for ROSCO2 is close to that of *α*-tocopherol mix rich in *γ* homolog, which shows a broad anticancer activity, including reducing estrogen receptor expression and increasing PPAR*γ* signalling in breast cancer cells [40]. Tocopherols are of course also powerful antioxidants and induce an antioxidant response by upregulating Nrf2 and transcription of its target genes [41]. The lack of influence of the tested oils on the production of ROS and NO in A549 at low concentrations of the emulsions reveals the advantage of the antioxidant effect of oils (rich in tocopherols and other antioxidants). Notwithstanding, in each line at the highest concentrations of oils, the oxidative stress and NO production were strongly expressed, especially under the influence of ROSCO2. The carotenoids possessing provitamin A activity and present in raspberry oils, greatly pronounced in the oil extracted by SCO2, e.g., *α*-carotene, *β*-carotene, and *β*-cryptoxanthin, may be potential agents in biological interference in cancer. Gloria et al. [17] observed inhibition of proliferation of breast cancer cell line MCF7 after treatment with *β*-carotene at 2.5 and 5 *μ*M for 48 h and found that *β*-carotene induced programmed cell death. From studies conducted on different cell lines (gastric cancer cell lines and B-cell lymphoma), it has been observed that treatment with *β*-carotene at a concentration exceeding physiological levels (100 *μ*M) increased the expression of p53 (proapoptotic protein) and decreased antiapoptotic BCL-2 [16]. Overall, the present studies demonstrate complex combinations of compounds that may modestly advance the therapy of cancer. Raspberry oil is an interesting example of such combination already existing in nature, and the way of its recovery may justify its chemopreventive potential.

A growing problem in cancer treatment is the increased resistance to commonly used cytostatics, including doxorubicin. Treatment of patients with chemotherapy becomes ineffective and often only results in the side effects of cytostatics and disease progression [42]. Significantly lower levels of free oxygen radicals (ROSs) are observed in cytostatic-resistant tumors [2]. Accordingly, damage to the DNA strand, and hence the death of cancer cells due to an increase in ROS or RNS, is significantly reduced [43]. As we found in our research, the raspberry oil emulsions were shown to possess good ability to propagate the generation of free oxygen radicals and NO. And especially ROSCO2 stimulates the generation of radicals in cultures of cells resistant to doxorubicin. The observed effects encourage further study to assess the synergistic effect of ROSCO2 with cytostatic.

The results of the calculated correlations may suggest that raspberry oil emulsions affect the NO-dependent mechanism of DNA strand damage in LoVo and MCF7 cells for both doxorubicin-resistant and sensitive lines. This is especially important when the exocytosis of drugs is reduced either by lysosomal or P-glycoprotein action [44, 45]. In the next stage of the research, it is planned to investigate the effect of the studied oils in combination with doxorubicin on cell cultures—evaluating the impact of reducing drug exocytosis and increasing the effectiveness of chemotherapy. Of course, an important aspect is the appropriate preparation of a therapy containing plant compounds that can undergo the first-pass metabolism. Therefore, an important aspect is the development of targeted therapies in the treatment of cancer.

## 5. Conclusions

Our study revealed many differences in the response of cell lines to the treatment of raspberry oils obtained by two different methods: extraction by supercritical CO_2_ and cold pressed. These data suggest that the way of oil manufacturing is important in terms of chemical characteristics, but what is also essential, may represent a cell suppression potential which may differ regarding the type of tumor. It is also important to conduct further investigations to determine the limitations for some other types of cancer. This study also showed that raspberry oil obtained by SCO2, due to its rich composition, may act in favor of chemoprevention of colon and breast cancers. We also observed that the metabolic activity of colon and breast cancer cell lines may be sensitive to the n-6/n-3 ratio.

Overall, the present study demonstrates the suppressive activity of raspberry oil, and it is the first study to suggest a role of raspberry oil in cancer prevention and/or therapy. The ability to increase the formation of radicals (NO and ROS), especially in doxorubicin cell lines, indicates possible adjunctive therapeutic applications, but this hypothesis needs further clarification.

## Figures and Tables

**Table 1:** Chemical characteristics of raspberry oil obtained by supercritical carbon dioxide extraction (ROSCO2) and cold pressed (ROCOP); statistically significant differences between oils (*p* < 0.05) are marked in bold.

	ROSCO2	ROCOP	*p*
Fatty acid composition (%)			
Palmitic acid	3.98 ± 0.18	5.37 ± 0.17	**0.00331**
Stearic acid	1.37 ± 0.15	2.58 ± 0.05	**0.00085**
Oleic acid (OA)	15.55 ± 0.85	26.38 ± 0.20	**0.00016**
Linoleic acid (LA)	50.78 ± 0.14	53.25 ± 0.24	**0.00108**
*α*-Linolenic acid (ALA)	27.22 ± 1.01	11.05 ± 0.11	**0.00008**
Others	1.10 ± 0.04	0.61 ± 0.03	**0.00047**

∑ SFA	6.24 ± 0.30	8.42 ± 0.22	**0.00229**
∑ MUFA	15.76 ± 0.86	27.28 ± 0.18	**0.00015**
∑ PUFA	78.00 ± 1.15	64.29 ± 0.23	**0.00021**
n-6 : n-3	1.87 ± 0.14	4.82 ± 0.06	**0.00001**

Phytosterols (mg/kg)			
Campesterol	616.38 ± 465.99	274.29 ± 46.35	**0.01773**
Stigmasterol	128.48 ± 7.74	147.63 ± 3.70	**0.09433**
*β*-Sitosterol	15020.17 ± 693.51	4082.44 ± 476.42	**0.00295**
*Δ*5-Avenasterol	2266.62 ± 161.28	595.91 ± 31.29	**0.00480**
Cycloartenol	445.99 ± 5.46	220.96 ± 12.34	**0.00179**
*Δ*^7^-Avenasterol	117.69 ± 1.25	54.82 ± 7.25	**0.00480**
24-Methylene-cycloartenol	1272.06 ± 42.71	432.04 ± 13.73	**0.00142**
Citrostadienol	221.73 ± 19.25	260.80 ± 60.45	0.47480
*β*-Amyrin (mg/kg)	181.14 ± 45.77	319.08 ± 35.04	0.16469
Total	20562.28 ± 919.47	6631.60 ± 251.00	**0.00325**

Squalene (mg/kg)	125.16 ± 0.62	110.68 ± 0.86	0.35420

Tocopherols (mg/kg)			
*α*-Tocopherol	513.37 ± 22.16	321.24 ± 15.02	**0.00128**
*γ*-Tocopherol	1979.98 ± 120.17	550.16 ± 42.21	**0.00026**
*δ*-Tocopherol	824.15 ± 11.10	234.06 ± 0.29	**0.00000**
Total	3317.50 ± 109.11	1105.26 ± 7.43	**0.00005**

Carotenoids (mg/kg)			
All-transneoxanthin	-	5.79 ± 1.02	-
All-transzeaxanthin	1329.05 ± 183.59	97.87 ± 11.06	**0.01017**
All-translutein	82.48 ± 5.90	51.55 ± 4.13	0.17910
All-trans-*α*-cryptoxanthin	255.32 ± 15.49	48.14 ± 1.42	**0.00014**
All-trans-*β*-cryptoxanthin	205.27 ± 26.38	10.75 ± 0.17	**0.00907**
Fucoxanthin	70.40 ± 11.19	-	-
All-trans-*α*-carotene	373.87 ± 19.70	27.30 ± 0.39	**0.00161**
All-trans-*β*-carotene	159.44 ± 12.48	12.49 ± 1.89	**0.00367**
Total	2475.85 ± 443.02	235.41 ± 26.26	**0.01906**

Phenolic compounds (mg/kg)			
4-Hydroxybenzoic acid	4.57 ± 0.038	0.05 ± 0.03	**0.00007**
Vanillic acid	2.88 ± 0.37	0.80 ± 0.21	**0.01382**
4-Hydroxybenzaldehyde	11.57 ± 1.53	3.37 ± 0.61	**0.03244**
Vanillin	16.32 ± 0.95	1.69 ± 0.10	**0.00184**
Sinapaldehyde	3.07 ± 0.15	0.22 ± 0.02	**0.00224**
*t*-Ferulic acid	1.06 ± 0.036	0.59 ± 0.02	**0.00676**
*Iso*ferulic acid	2.05 ± 0.09	-	-
Total	41.52 ± 2.98	6.76 ± 0.48	**0.00404**

**Table 2:** IC_50_ values (concentration that causes 50% decrease in cell growth) of oil emulsions from raspberry oils extracted by supercritical CO_2_ (ROSCO2) and cold pressed (ROCOP) against different cell lines in inhibition of metabolic activity (MTT) assay.

Cell line	IC_50_ values (%)		*p* value
	ROSCO2	ROCOP	
LoVo	17.3 (3.4)	10.3 (5.2)	0.03
LoVo/DX	9.6 (6.3)	6.9 (5.4)	0.48
MCF7	49.5 (8.4)	51.7 (10.1)	0.72
MCF7/DX	24.9 (2.8)	29.4 (3.1)	0.04
A549	25.9 (17.6)	12.7 (15.6)	0.25
NHDF	51.9 (3.5)	88.4 (3.7)	0.0001

MCF7: breast cancer cell line; MCF7/DX: doxorubicin-resistant breast cancer cell line; A549: lung cancer cell line; NHDF: normal human dermal fibroblast. The results are presented as mean (SD).

**Table 3:** IC_50_ values (concentration that causes 50% decrease in cell growth) of oil emulsions from raspberry oils extracted by supercritical CO_2_ (ROSCO2) and cold pressed (ROCOP) against different cell lines determined with the SRB assay. The results are presented as mean (SD).

Cell line	IC_50_ values (%)		*p* value
	ROSCO2	ROCOP	
LoVo	15.0 (2.5)	23.1 (3.4)	0.0026
LoVo/DX	17.1 (2.2)	18.7 (3.0)	0.36
MCF7	69.4 (7.3)	35.5 (8.0)	0.0001
MCF7/DX	48.3 (2.2)	52.2 (2.8)	0.04
A549	14.5 (2.8)	14.4 (2.7)	0.96
NHDF	Nontoxic	57.0 (2.4)	N/A

LoVo: colon adenocarcinoma cell line; LoVo/DX: doxorubicin-resistant colon adenocarcinoma cell line; MCF7: breast cancer cell line; MCF7/DX: doxorubicin-resistant breast cancer cell line; A549: lung cancer cell line; NHDF: normal human dermal fibroblast; N/A: not applicable.

**Table 4:** Statistically significant correlation coefficients between DNA damage and levels of reactive oxygen species (ROS) and nitric oxide (NO).

Cell line	Oil type	ROS level vs. DNA damage	NO level vs. DNA damage
LoVo	ROSCO2	0.52	0.87
	ROCOP	0.89	0.7

LoVo/DX	ROSCO2	-0.34	0.76
	ROCOP	0.32	0.7

MCF7	ROSCO2	0.5	0.61
	ROCOP	-0.29	0.52

MCF7/DX	ROSCO2	0.64	0.72
	ROCOP	0.56	0.71

A549	ROSCO2	-	-0.36
	ROCOP	0.64	0.76

LoVo: colon adenocarcinoma cell line; LoVo/DX: doxorubicin-resistant colon adenocarcinoma cell line; MCF7: breast cancer cell line; MCF7/DX: doxorubicin-resistant breast cancer cell line; A549: lung cancer cell line; ROSCO2: raspberry oil extracted by supercritical CO2; ROCOP: raspberry oil cold-pressed.

## Data Availability

The data used to support the findings of this study are available from the corresponding author upon request.

## References

[1] Cheng M.-H., Huang H.-L., Lin Y.-Y. (2019). BA6 induces apoptosis via stimulation of reactive oxygen species and inhibition of oxidative phosphorylation in human lung cancer cells. *Oxidative Medicine and Cellular Longevity*.

[2] Piasny J., Wiatrak B., Dobosz A., Tylińska B., Gębarowski T. (2020). Antitumor activity of new olivacine derivatives. *Molecules*.

[3] Moreira H., Szyjka A., Paliszkiewicz K., Barg E. (2019). Prooxidative activity of celastrol induces apoptosis, DNA damage, and cell cycle arrest in drug-resistant human colon cancer cells. *Oxidative Medicine and Cellular Longevity*.

[4] Oomah B. D., Ladet S., Godfrey D. V., Liang J., Girard B. (2000). Characteristics of raspberry (Rubus idaeus L.) seed oil. *Food Chemistry*.

[5] Pieszka M., Migdał W., Gąsior R. (2015). Native oils from apple, blackcurrant, raspberry, and strawberry seeds as a source of polyenoic fatty acids, tocochromanols, and phytosterols: a health implication. *Journal of Chemistry*.

[6] Radočaj O., Vujasinović V., Dimić E., Basić Z. (2014). Blackberry (Rubus fruticosus L.) and raspberry (Rubus idaeus L.) seed oils extracted from dried press pomace after longterm frozen storage of berries can be used as functional food ingredients. *European Journal of Lipid Science and Technology*.

[7] Yang B., Ahotupa M., Määttä P., Kallio H. (2011). Composition and antioxidative activities of supercritical CO_2_-extracted oils from seeds and soft parts of northern berries. *Food Research International*.

[8] Pieszka M., Tombarkiewicz B., Roman A., Migdał W., Niedziółka J. (2013). Effect of bioactive substances found in rapeseed, raspberry and strawberry seed oils on blood lipid profile and selected parameters of oxidative status in rats. *Environmental Toxicology and Pharmacology*.

[9] Fotschki B., Jurgoński A., Juśkiewicz J., Zduńczyk Z. (2015). Dietary supplementation with raspberry seed oil modulates liver functions, inflammatory state, and lipid metabolism in rats. *The Journal of Nutrition*.

[10] González-Fernández M. J., Ortea I., Guil-Guerrero J. L. (2020). *α*-Linolenic and *γ*-linolenic acids exercise differential antitumor effects on HT-29 human colorectal cancer cells. *Toxicology Research*.

[11] Chamberland J. P., Moon H.-S. (2015). Down-regulation of malignant potential by alpha linolenic acid in human and mouse colon cancer cells. *Familial Cancer*.

[12] Desai S. J., Prickril B., Rasooly A. (2018). Mechanisms of phytonutrient modulation of cyclooxygenase-2 (COX-2) and inflammation related to cancer. *Nutrition and Cancer*.

[13] Das Gupta S., Suh N. (2016). Tocopherols in cancer: an update. *Molecular Nutrition & Food Research*.

[14] Abraham A., Kattoor A. J., Saldeen T., Mehta J. L. (2019). Vitamin E and its anticancer effects. *Critical Reviews in Food Science and Nutrition*.

[15] Milani A., Basirnejad M., Shahbazi S., Bolhassani A. (2017). Carotenoids: biochemistry, pharmacology and treatment. *British Journal of Pharmacology*.

[16] Niranjana R., Gayathri R., Nimish Mol S. (2015). Carotenoids modulate the hallmarks of cancer cells. *Journal of Functional Foods*.

[17] Gloria N. F., Soares N., Brand C., Oliveira F. L., Borojevic R., Teodoro A. J. (2014). Lycopene and beta-carotene induce cell-cycle arrest and apoptosis in human breast cancer cell lines. *Anticancer Research*.

[18] Blanco-Vaca F., Cedó L., Julve J. (2019). Phytosterols in cancer: from molecular mechanisms to preventive and therapeutic potentials. *Current Medicinal Chemistry*.

[19] Maqbool W., Hobson P., Dunn K., Doherty W. (2017). Supercritical carbon dioxide separation of carboxylic acids and phenolics from bio-oil of lignocellulosic origin: understanding bio-oil compositions, compound solubilities, and their fractionation. *Industrial & Engineering Chemistry Research*.

[20] Xu X., Dong J., Mu X., Sun L. (2011). Supercritical CO2 extraction of oil, carotenoids, squalene and sterols from lotus (Nelumbo nucifera Gaertn) bee pollen. *Food and Bioproducts Processing*.

[21] Milala J., Grzelak-Błaszczyk K., Sójka M., Kosmala M., Dobrzyńska-Inger A., Rój E. (2018). Changes of bioactive components in berry seed oils during supercritical CO2extraction. *Journal of Food Processing and Preservation*.

[22] Prescha A., Swiedrych A., Biernat J., Szopa J. (2001). Increase in lipid content in potato tubers modified by 14-3-3 gene overexpression. *Journal of Agricultural and Food Chemistry*.

[23] Shukla V., Dutta P., Artz W. (2002). Camelina oil and its unusual cholesterol content. *Journal of the American Oil Chemists' Society*.

[24] Fromm M., Bayha S., Kammerer D. R., Carle R. (2012). Identification and quantitation of carotenoids and tocopherols in seed oils recovered from different Rosaceae species. *Journal of Agricultural and Food Chemistry*.

[25] Pirisi F. M., Angioni A., Cabras P. (1997). Phenolic compounds in virgin olive oils I. Low-wavelength quantitative determination of complex phenols by high-performance liquid chromatography under isocratic elution. *Journal of Chromatography A*.

[26] Skorkowska-Telichowska K., Hasiewicz-Derkacz K., Gębarowski T. (2016). Emulsions made of oils from seeds of GM flax protect V79 cells against oxidative stress. *Oxidative Medicine and Cellular Longevity*.

[27] Kulbacka J., Choromańska A., Drąg-Zalesińska M. (2020). Proapoptotic activity induced by photodynamic reaction with novel cyanine dyes in caspase-3-deficient human breast adenocarcinoma cell lines (MCF/WT and MCF/DX). *Photodiagnosis and Photodynamic Therapy*.

[28] Grajzer M., Wiatrak B., Gębarowski T. (2021). Chemistry, oxidative stability and bioactivity of oil extracted from Rosa rugosa (Thunb.) seeds by supercritical carbon dioxide. *Food Chemistry*.

[29] Wakulik K., Wiatrak B., Szczukowski Ł. (2020). Effect of novel pyrrolo [3, 4-d] pyridazinone derivatives on lipopolysaccharide-induced neuroinflammation. *International Journal of Molecular Sciences*.

[30] Šućurović A., Vukelić N., Ignjatović L., Brčeski I., Jovanović D. (2009). Physical-chemical characteristics and oxidative stability of oil obtained from lyophilized raspberry seed. *European Journal of Lipid Science and Technology*.

[31] Brandão D., Ribeiro L. (2018). Dietary fatty acids modulation of human colon cancer cells: mechanisms and future perspectives. *International Journal of Food Sciences and Nutrition*.

[32] Lu X., Yu H., Ma Q., Shen S., Das U. N. (2010). Linoleic acid suppresses colorectal cancer cell growth by inducing oxidant stress and mitochondrial dysfunction. *Lipids in Health and Disease*.

[33] Zhang C., Yu H., Ni X., Shen S., Das U. N. (2015). Growth inhibitory effect of polyunsaturated fatty acids (PUFAs) on colon cancer cells via their growth inhibitory metabolites and fatty acid composition changes. *PLoS One*.

[34] Wiggins A. K. A., Mason J. K., Thompson L. U. (2015). Growth and gene expression differ over time in alpha-linolenic acid treated breast cancer cells. *Experimental Cell Research*.

[35] Zhang T., Liu R., Chang M., Jin Q., Zhang H., Wang X. (2020). Health benefits of 4, 4-dimethyl phytosterols: an exploration beyond 4-desmethyl phytosterols. *Food & Function*.

[36] Constantinou C., Neophytou C. M., Vraka P., Hyatt J. A., Papas K. A., Constantinou A. I. (2012). Induction of DNA damage and caspase-independent programmed cell death by vitamin E. *Nutrition and Cancer*.

[37] Campbell S. E., Musich P. R., Whaley S. G. (2009). Gamma tocopherol upregulates the expression of 15-S-HETE and induces growth arrest through a PPAR gamma-dependent mechanism in PC-3 human prostate cancer cells. *Nutrition and Cancer*.

[38] Schmidt M. V., Brüne B., von Knethen A. (2010). The nuclear hormone receptor PPAR*γ* as a therapeutic target in major diseases. *TheScientificWorldJOURNAL*.

[39] Granci V., Cai F., Lecumberri E., Clerc A., Dupertuis Y. M., Pichard C. (2013). Colon cancer cell chemosensitisation by fish oil emulsion involves apoptotic mitochondria pathway. *British Journal of Nutrition*.

[40] Smolarek A. K., Suh N. (2011). Chemopreventive activity of vitamin E in breast cancer: a focus on *γ*-and *δ*-Tocopherol. *Nutrients*.

[41] Smolarek A. K., So J. Y., Thomas P. E. (2013). Dietary tocopherols inhibit cell proliferation, regulate expression of ER*α*, PPAR*γ*, and Nrf2, and decrease serum inflammatory markers during the development of mammary hyperplasia. *Molecular Carcinogenesis*.

[42] Hua X., Sun Y., Chen J. (2019). Circular RNAs in drug resistant tumors. *Biomedicine & Pharmacotherapy*.

[43] Russell E., O’Sullivan E., Miller C., Stanicka J., McCarthy F., Cotter T. (2014). Ellipticine derivative induces potent cytostatic effect in acute myeloid leukaemia cells. *Investigational New Drugs*.

[44] Zhitomirsky B., Assaraf Y. G. (2017). Lysosomal accumulation of anticancer drugs triggers lysosomal exocytosis. *Oncotarget*.

[45] Sun Y., Zhang J., Yin H., Yin J. (2020). MicroRNA-mediated suppression of P-glycoprotein by quantum dots in lung cancer cells. *Journal of Applied Toxicology*.

